# A Novel Foam-Forming Hydrogel Containing Antibiotics Reduces the Degree of Bacterial Colonization and Improves Wound Healing in Thermal Burns When Compared to Silver Sulfadiazine

**DOI:** 10.1093/milmed/usag049

**Published:** 2026-08-06

**Authors:** Ross I Donaldson, Diane Goldenberg, Todd L Graham, David A Tanen, Nely N Cristerna, Oliver J Buchanan, John S Cambridge, Jonathan K Armstrong, Fiona Latifi, Juliana Tolles, Maja Engler, James D Ross

**Affiliations:** Critical Innovations LLC, Los Angeles, CA 90260, United States; Department of Emergency Medicine, David Geffen School of Medicine at UCLA, Los Angeles, CA 90095, United States; Department of Emergency Medicine, Harbor-UCLA Medical Center, Torrance, CA 90509, United States; Critical Innovations LLC, Los Angeles, CA 90260, United States; Benchmark Biotech LLC, Portland, OR 97232, United States; Department of Emergency Medicine, David Geffen School of Medicine at UCLA, Los Angeles, CA 90095, United States; Department of Emergency Medicine, Harbor-UCLA Medical Center, Torrance, CA 90509, United States; Critical Innovations LLC, Los Angeles, CA 90260, United States; Critical Innovations LLC, Los Angeles, CA 90260, United States; Critical Innovations LLC, Los Angeles, CA 90260, United States; Critical Innovations LLC, Los Angeles, CA 90260, United States; Critical Innovations LLC, Los Angeles, CA 90260, United States; Department of Emergency Medicine, David Geffen School of Medicine at UCLA, Los Angeles, CA 90095, United States; Department of Emergency Medicine, Harbor-UCLA Medical Center, Torrance, CA 90509, United States; Benchmark Biotech LLC, Portland, OR 97232, United States; Benchmark Biotech LLC, Portland, OR 97232, United States

## Abstract

**Introduction:**

This study compared a novel antibiotic hydrogel burn dressing to non-antibiotic hydrogel vehicle, standard of care (silver sulfadiazine), and untreated control in a 7-day porcine partial-thickness (second-degree) thermal burn model to assess for wound healing properties both in the presence and absence of bacterial inoculation.

**Materials and Methods:**

Nineteen swine were enrolled and 18 completed Day 7 analysis. One was euthanized on Day 6 because of an unrelated injury. Eight burn wounds were created on each swine, with half of the wounds inoculated with *Staphylococcus aureus* and *Pseudomonas aeruginosa*. Wound treatment was randomized to 4 groups: foam-based hydrogel spray containing antibiotics (i.e., vancomycin and tobramycin), the same hydrogel vehicle without antibiotics, silver sulfadiazine, and no intervention. Observations and primary and secondary outcomes were assessed only on Day 7. A blinded pathologist graded the specimens for bacterial colonization and wound healing.

**Results:**

The antibiotic hydrogel demonstrated significantly less bacterial colonization when compared to hydrogel vehicle, silver sulfadiazine, and control (odds ratios of 0.02, 0.02, and 0.01, respectively). The antibiotic hydrogel also demonstrated statistically significant benefits to wound healing when compared to hydrogel vehicle, silver sulfadiazine, and control. Wound healing parameters were factors of histopathology: necrosis depth/length, ulceration, neutrophilic/granulocytic infiltrate, subcutis granulocytic/lymphocytic infiltrates, subcutis fibrosis, congestion, hemorrhage, and edema.

**Conclusions:**

In a 7-day porcine partial thickness burn model, an antibiotic hydrogel foam reduced the degree of bacterial colonization and improved wound healing when compared to non-antibiotic hydrogel vehicle, standard of care, and control. Strengths of this study include the randomization of multiple treatment conditions, blinded pathology, and the inclusion of both inoculated and non-inoculated wounds with clinically relevant bacterial strains. Limitations include a modest sample size and limited quantitative bacteriology. A lightweight, portable foam-based antibiotic hydrogel spray may improve the early, prehospital treatment of thermal burns. Future work may assess use over longer time periods and body surface areas and increased quantitative microbiology.

## INTRODUCTION

Thermal burns and subsequent infections thereof are a significant threat to American troops and occur both during training and on the battlefield.^1^ They also account for thousands of occupational and home injuries for civilians each year.^2^^,^^3^ Burn wound healing is often hindered by infection, particularly on the front lines of combat. Harmful bacterial colonization remains the primary cause of mortality in burn victims, while the standard of care (silver sulfadiazine) has not advanced since the early 1970s.^4^^,^^5^ Advancement in the care and treatment of burns is thus urgently needed.

In austere and far-forward environments, burn injuries may be contaminated at the time of injury and immediate wound decontamination may be impractical. In contrast, many civilian burns can be irrigated or cleansed before topical therapy application. Silver sulfadiazine is commonly used as prophylaxis against subsequent contamination. This study was designed accordingly to model simultaneous injury and contamination by inoculating wounds immediately after burn creation and applying treatment shortly thereafter. It was hypothesized that an antibiotic-containing thermoreversible hydrogel foam (HA) would reduce bacterial colonization and improve histologic markers of wound healing at Day 7 compared with vehicle-only hydrogel (HV), silver sulfadiazine (SS), and no treatment control (CO). This hypothesis was tested using randomized wound-level allocation within animals and blinded histopathologic assessment.

Hydrogels are water-based polymers that act as a moist, protective barrier to promote wound healing and provide non-adherent pain relief.^6^^,^^7^ They are widely available as pads, gels, or sprays and have been shown to significantly improve wound healing in thermal burns.^8^ Hydrogels are also a unique drug vehicle in which antibiotics can be stably dissolved and directly applied to the wound to reduce bacterial colonization and prevent infection. Improvements in hydrogel technology and their use for treating thermal wounds represent a major opportunity for innovation in the field of burn care and treatment.

In a previous pilot study, it was demonstrated that a hydrogel foam product containing vancomycin and tobramycin (Critical Innovations LLC, CA, United States) decreased necrosis and bacterial growth when compared to no treatment.^9^ In the preceding swine model of thermal injury with bacterial inoculation, the hydrogel treatment performed comparably to silver sulfadiazine, while being easier to remove for repeat clinical examinations.

In this larger follow-up study, we utilized an improved swine model to compare the amount of bacterial colonization and degree of wound healing at 7 days between 4 different treatment groups: thermoreversible hydrogel foam containing the antibiotics vancomycin and tobramycin (HA), the same foam-based hydrogel vehicle alone without antibiotics (HV), silver sulfadiazine (SS; a standard of care comparator), and no treatment control (CO).^10^

## METHODS

### Regulatory Information

Approval was obtained from the Legacy Research Institute’s Institutional Animal Care and Use Committee and the US Army’s Animal Care and Use Review Office (143-2023 and MT21002.002.e001, respectively). Experiments were conducted in a facility that is authorized by the Association for Assessment and Accreditation of Laboratory Animal Care. Animals were used in accordance with The Guide for the Care and Use of Laboratory Animals. The use of animals for this research complied with all ARRIVE guidelines.^11^

### Materials

Lyophilized bacteria were obtained from American Type Culture Collection (ATCC); Manassas, VA, USA) and consisted of *Staphylococcus aureus* (*S. aureus;* ATCC 12600) and *Pseudomonas aeruginosa* (*P. aeruginosa*; ATCC 27853), 2 of the most common pathological bacteria causing infections in burn injuries.^12^ They were prepared to a concentration of 1.8E8–7.3E8 colony forming units (CFU) per mL. The bacterial counts were confirmed by serial dilution, plating, and counting.

Wound dressings were prepared as follows: Canisters of HA and HV were obtained from Critical Innovations LLC, CA, United States. HA consisted of a thermoreversible hydrogel foam containing antibiotics. The antibiotics vancomycin and tobramycin (Tecoland Corporation, CA, United States) were added to the hydrogel to achieve a concentration of 0.35% and 0.07%, respectively. HV consisted of the same foam-based hydrogel without antibiotics and acted as a vehicle-only comparison. SS consisted of silver sulfadiazine (Medline Industries, GA, United States), representing the standard of care. CO involved no intervention and represented the control group.

### Animal Sedation and Preparation

Nineteen male swine (*Sus scrofa*) were obtained from a single source vendor (Premier BioSource, CA, United States), acclimated to their surroundings before the study initiation, and weighed. Animals were pre-medicated with 0.2 mg/kg meloxicam orally. They were then sedated with 4-8 mg/kg Telazol (Zoetis, NJ, United States) and 0.04 mg/kg of atropine via intramuscular injection. The animals were intubated and mechanically ventilated after induction with isoflurane and remained on isoflurane at 1%-5% to maintain adequate anesthesia. In addition, animals received 0.005-0.01 mg/kg of buprenorphine as an analgesic. Percutaneous access to the external jugular vein was also achieved and baseline blood was withdrawn for analysis.

### Burn Wound Creation and Bacterial Inoculation

Eight burn wounds were created per animal using a previously described burn device that consists of a fixed-force 3-cm diameter cylindrical aluminum block.^9^^,^^13^ After hair removal and surgical scrub (betadine and 70% ethanol), the device was heated to 100 °C and then, for each wound, pressed against the paraspinal region of the pig’s dorsum for 20 seconds to create the partial thickness burns. Four wounds were made dorsally on the contralateral sides of the spine, resulting in 8 wounds per animal. After burn creation, the prepared bacterial solution for each strain was directly pipetted onto 4 wound sites per animal and dispersed from the center to the outside edge of the burn wound using an inoculation loop. The other half of the wounds were treated with an equal volume of phosphate-buffered saline (PBS) vehicle, as a non-infected comparison.

### Intervention

Ten minutes after bacterial inoculation, each wound was randomized into 1 of 4 study groups: HA (hydrogel ­antibiotic), HV (hydrogel vehicle), SS (silver sulfadiazine), and CO (no treatment control). The 10-minute inoculation-to-treatment time was standardized across treatment groups. HA and HV were applied in accordance with the HA product’s Instructions for Use (IFU), which instructed the user to deploy the foaming hydrogel over the wound. SS was administered as directed per the silver sulfadiazine IFU. The masses of HA, HV, and SS applied were documented. CO animals received no intervention (control). After application, all wounds were then covered with a Tegaderm (3M) dressing to prevent bacterial or product cross-contamination. For groups receiving treatment (HV, HA, and SS), treatment was reapplied daily for 7 days.

### Animal Recovery and Monitoring

Blood samples were collected at baseline (before burn creation), and at 1-hour, 1-day, 3-day, and 7-day time-points post treatment. Samples were analyzed for complete blood counts, chemistry analysis, and antibiotic plasma levels. Vancomycin and tobramycin concentrations were determined using Liquid Chromatography tandem Mass Spectrometry (LC-MS/MS) method by a third-party laboratory (Alturas Analytics, Inc., ID, United States).

Animals were observed for 1 hour after burn wound creation and initial treatment. Animals were then weaned off anesthesia, extubated, and returned to their housing. For continued analgesia, all animals received a transdermal fentanyl patch (Duragesic, Janssen, Beerse, Belgium) which released 5 μg/kg/hour for 72 hours. Wounds were inspected and redressed daily. One animal was humanely euthanized on Day 6 because of an idiopathic hindlimb injury determined to be unrelated to study aims. Eighteen animals were followed to the scheduled end of study (Day 7). At the end of the 7-day observation period, all remaining animals were humanely euthanized with 1 mL/10 lbs of Euthanasia Solution (VetOne, ID, United States) while under general anesthesia.

### Tissue Analysis

Upon euthanasia of the animals, dressings were removed, and wounds were swabbed for microbiological identification. Biopsied tissue was fixed in 10% neutral buffered formalin, embedded in paraffin, sectioned, and stained with hematoxylin-eosin (H&E). Using a previously published grading scale for measuring the level of bacterial presence and wound healing, a pathologist blinded to study treatments assessed the samples for evidence of infection, inflammation, and wound healing.^9^ The assessment, which has been previously described, consisted of assigning a score on a 5-category ordinal scale ranging from 0 to 4, in which higher grades denote less desirable outcomes.

### Sample Size Justification and Analysis

The primary outcome was histopathologic grading of bacterial presence (graded 0-4). An *a priori* sample size calculation, with a cumulative logistic regression as the prespecified primary analysis method, was performed and a sample size of 18 animals, with 8 burn wounds per animal (total of 144 burn wounds), was considered to detect an odds ratio of 0.7. We also prespecified a non-inferiority margin of 1.5 on the log-odds scale, for example, log(1.5), for the planned non-inferiority analysis with respect to SS.

Because many of the samples were missing some portion of the serocellular crust and grading of bacterial presence on a partial surface would not have been representative, bacterial presence was ultimately graded by the reviewing pathologist as a dichotomous outcome (0,1). Therefore, the analytic plan was updated to analyze the primary outcome with a generalized estimating equation, using a logit link function, with fixed effects for treatment (control as the reference category), inoculated versus not inoculated, and a random effect by animal (R package “brms”). Bayesian regression methodology was selected for the analysis to attain greater stability of parameter estimates in the setting of a small sample size. A normal prior distribution, with a mean of 0 and standard deviation of 3, was placed on the treatment effect associated with each factor, to reflect that treatment effect odds ratios less than 0.01 or greater than 100 are rarely observed. The same prior was used for all analyses below.

For the non-inferiority comparison of HA to SS, the same model was fitted but silver sulfadiazine was used as the reference category for treatment. We selected a critical value cutoff of 99.3% for the posterior probability that the odds ratio is less than 1 to account for multiple testing. A sensitivity analysis was performed without a random effect for clustering by animal.

For the analysis of all the secondary outcomes comparing histologic analysis of wound healing, apart from re-epithelialization, a Bayesian cumulative logistic regression model was fitted with fixed effects for treatment and inoculated versus not inoculated and a random effect for animal (R package “brms”). A model could not be fit to the re-epithelialization outcome because of limited data and insufficient representation across outcome categories.

An exploratory analysis of the quantitative bacterial growth outcomes was performed for both *P. aeruginosa* and *S. aureus*. Bacterial growth was categorized into 3 ordered classifications: no growth, some growth but less than “too numerous to count,” and “too numerous to count.” Separately, for each of the 2 species of bacteria, a Bayesian generalized estimating equation was fitted using a cumulative logit link function with fixed effects for treatment and quantity of bacterial inoculum (for non-inoculated wounds, the quantity of bacterial inoculum was assigned as 0 in the regression analysis) and a random effect for clustering by animal.

## RESULTS

Eighteen swine weighing 47.6 ± 1.7 kg were studied. The animals averaged an age of 96 ± 6 days. The average mass of HA applied to each wound was 0.625 g. The average masses of vancomycin and tobramycin applied to each wound were 2.18 mg and 0.456 mg, respectively. The *S. aureus* inoculum averaged 1.96 × 10^8^ CFU per wound and the *P. aeruginosa* inoculum averaged 6.19 × 10^8^ CFU per wound. Within each animal, all infected wounds received the same bacterial concentration. There were no complications or adverse reactions to any of the applied treatments.

**Figure 1.**
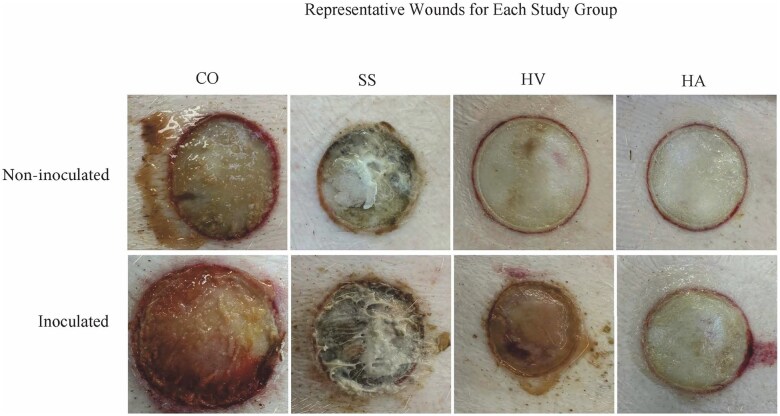
Representative Day-7 wound images by treatment and inoculation status. Shown are representative photographs of non-inoculated and inoculated wounds for each treatment group at Study Day 7. Across both wound types, wounds treated with HA demonstrate visibly greater healing by the end of the study compared with other treatments. (A) CO, non-inoculated; (B) CO, inoculated; (C) SS, non-inoculated; (D) SS, inoculated; (E) HV, non-inoculated; (F) HV, inoculated; (G) HA, non-inoculated; (H) HA, inoculated. Abbreviations: CO, control; HA, hydrogel with antibiotic; HV, hydrogel vehicle; SS, silver sulfadiazine.

Bloodwork and histopathologic specimens were obtained from all animals. Neither vancomycin nor tobramycin plasma levels were detected in any blood samples drawn throughout the study, with a lower limit of quantitation (LLOQ) of 50 ng/mL used for both antibiotics. Figure 1 depicts representative samples for each study group on Day 7 of the study.

The pathologist graded signs of infection, inflammation, and wound healing for non-inoculated and bacterially inoculated wounds from each treatment group (Figures S1 and S2). As shown in Table 1, histological examination revealed that HA demonstrated significantly lower levels of bacterial colonization in the wounds when compared to CO, SS, and HV. In contrast, Table S1 shows that HV has minimal effect when compared to CO and SS, with the credible intervals crossing 1.

**Table 1.** Primary Analysis: Bacterial Colonization on Histopathology

Comparison	Odds ratio	95% CI	Posterior Pr(OR < 1) (%)
HA vs. CO	0.01	4e–4-0.10	100
HA vs. SS	0.02	7e–4-0.22	100
HA vs. HV	0.02	6e–4-0.16	100
Inoculated vs. not inoculated	1.92	1.14-3.39	1.4
SS vs. CO	0.53	0.26-1.00	97

Evidence of reduced bacterial burden with HA versus standard comparators. Displayed are ORs (95% CIs) for histopathologic colonization; values **<**1 signal fewer bacteria with HA relative to CO, SS, and HV. Posterior probabilities [Pr(OR < 1)] quantify the strength of this effect. As expected, inoculated wounds show higher colonization than non-inoculated controls. Abbreviations: CI, confidence interval; CO, control; HA, hydrogel with antibiotic; HV, hydrogel vehicle; OR, odds ratio; Pr(OR < 1), posterior probability that OR < 1; SS, silver sulfadiazine.

**Figure 2.**
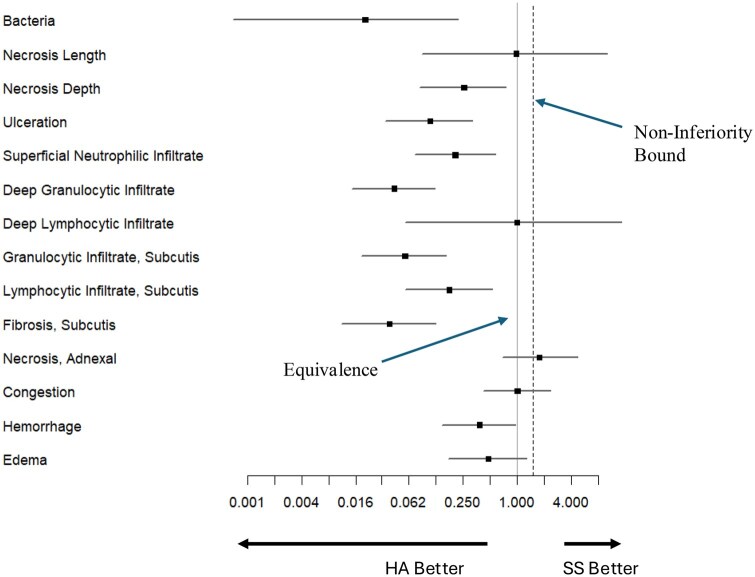
Forest plot of Day-7 histopathologic outcomes comparing HA versus SS. Forest plot displays effect estimates (points) with confidence intervals (horizontal bars) for outcomes listed on the y-axis (e.g., bacteria, necrosis length/depth, ulceration, superficial and deep inflammatory infiltrates, subcutis findings, congestion, hemorrhage, edema). The solid vertical line at 1.0 denotes equivalence; the dashed vertical line marks the prespecified non-inferiority bound. Estimates to the left favor HA, and to the right favor SS. Abbreviations: HA, hydrogel with antibiotic; SS, silver sulfadiazine.

Secondary outcomes for HA versus SS are shown in Figure 2. HA outperformed SS regarding bacterial presence, necrosis depth, ulceration, superficial neutrophilic infiltrate, deep granulocyte infiltrate, granulocytic infiltrate (subcutis), lymphocytic infiltrate (subcutis), fibrosis (subcutis), congestion, and hemorrhage.

**Figure 3.**
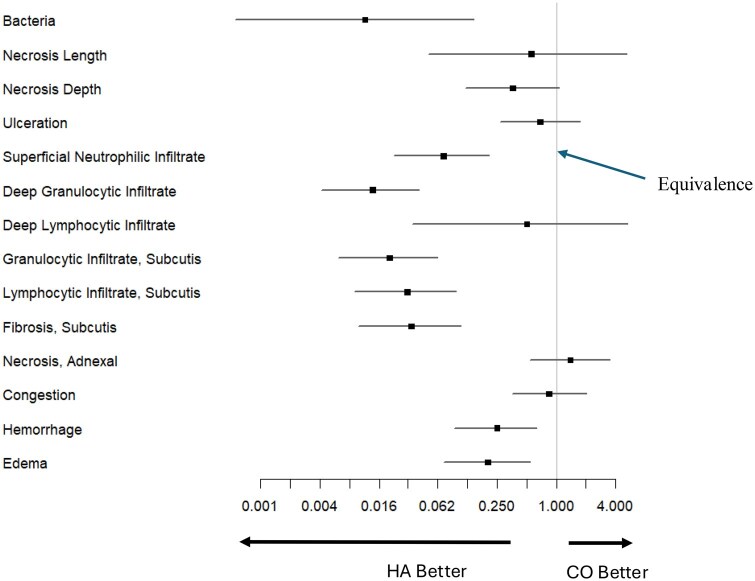
Forest plot of Day-7 histopathologic secondary outcomes comparing HA versus CO. Forest plot shows effect estimates (squares) with 95% confidence intervals (horizontal bars) for each outcome listed at left (bacteria; necrosis length and depth; ulceration; superficial and deep neutrophilic/granulocytic/lymphocytic infiltrates, including subcutis; fibrosis, subcutis; necrosis, adnexal; congestion; hemorrhage; edema). The vertical line at 1,000 marks equivalence; points to the left favor HA and to the right favor CO. Abbreviations: CO, control; HA, hydrogel with antibiotic.

Secondary outcomes for HA versus CO are shown in Figure 3. HA outperformed CO in the outcomes of bacterial presence, superficial neutrophilic infiltrate, deep granulocytic infiltrate, subcutaneous granulocytic infiltrate, subcutaneous lymphocytic infiltrate, subcutaneous fibrosis, hemorrhage, and edema. The following secondary outcomes also demonstrated a potential benefit for HA over CO, but were not statistically significant with a 95% confidence interval crossing an odds ratio of 1: ulceration, deep lymphocyte infiltrate, necrosis length, and tissue congestion. Only the point estimate for adnexal necrosis favored CO, although this also was not statistically significant with a 95% confidence interval crossing an odds ratio of 1. Analysis of the secondary outcomes was limited by substantial uncertainty in some estimates because of small sample sizes.

Results of the exploratory analyses for microbiological growth are shown in Tables S2 and S3. Overall, the data lacked strong posterior probabilities to confidently determine treatment effects. Exceptions included: for *S. aureus,* reduced bacterial growth when comparing HA to CO; for *S. aureus,* reduced bacterial growth when comparing SS to CO; for *P. aeruginosa*, increased bacterial growth when comparing HV to SS.

## DISCUSSION

This porcine study of thermal burns inoculated with gram-positive and gram-negative bacteria found that a foam-based hydrogel containing antibiotics led to a significant decrease in the rates of bacterial colonization when compared with the non-antibiotic foam-based hydrogel vehicle, silver sulfadiazine, or no treatment. The study also demonstrated improvement in secondary markers of wound healing when comparing HA against HV, SS, and CO.

These findings support the hypothesis that decreasing bacterial colonization enhances the skin’s ability to re-epithelialize and improves wound healing.^14^ In this experiment, the thermal burn model reliably induced epidermal, subdermal, and muscle injury as evidenced by observation and histological examination.^13^ Bacterial colonization with common wound pathogens was also successfully achieved by inoculating half the wounds using premixed bacterial cultures.

Topical antibiotics are widely considered efficacious and are found in many over-the-counter wound preparations.^15^ However, most of these products are aimed at minor wounds and include antibiotics that are toxic if absorbed in large quantities (e.g., neomycin sulfate, polymyxin B, bacitracin). This presents a problem for the treatment of burn wounds, which may cover large body surface areas and can result in the loss of protective skin epithelium, both of which potentially significantly increase drug absorption.

To date, silver products (e.g., silver sulfadiazine) have thus been the mainstay of burn infection topical prevention. However, in austere settings, repeated dressings changes and application and removal of topical agents that adhere to the wound surface may contribute to unnecessary patient discomfort or pain, particularly when analgesia is limited. Additional challenges include being able to evaluate the wound once treatment has been applied and limited efficacy. Thus, a topical product that overcomes these drawbacks, is more rapidly applied, and more easily removed would have numerous clinical benefits, especially in the field.

For the HA product, the purposeful use of antibiotics that are already FDA-approved for parenteral injection reduces the chances of significant safety side effects should the drugs be absorbed. The concentrations of antibiotics in HA are also lower than those typically found in available commercial products. Specifically, vancomycin in HA is at a concentration 0.35%, compared to the 5% in combination Mupirocin 2%/Vancomycin 5% Topical Ointment. Similarly, tobramycin in HA is at a concentration of 0.07%, compared to 0.3% in Tobramycin Ophthalmic Ointment. These lower concentrations may support safe application of HA over very large total body surface areas, which could be explored further in future studies.

Additionally, deployment of the antibiotics within this novel hydrogel foam may offer several clinical advantages as a burn management solution. Hydrogels provide a moist, protective barrier thought to improve wound healing.^6^^,^^16^^,^^17^ However, typical hydrogels can be challenging to deploy.^1^^,^^15^^,^^18^ This unique reverse-phase-shifting hydrogel foam can be more easily and rapidly applied to irregular and large-surface-area wounds. Additionally, its thermoreversible nature makes it much easier to remove than alternative treatments.

### Limitations

We selected a well-described swine model of thermal burn injury to test our hypothesis. Although thermal burns produced in this swine model have previously been reported to heal in a manner similar to human burns, caution is needed when extrapolating results from swine to human histopathology.^13^^,^^19^

This study included systemic (plasma) antibiotic testing of vancomycin and tobramycin, but did not detect either drug at 1-hour, 1-day, 3-day, and 7-day time-points post initial treatment, based on an LLOQ of 50 ng/mL for both antibiotics. Although this does demonstrate that there was not a significant systemic buildup of antibiotics over time, this study was not designed nor does it provide sufficient data for a full pharmacokinetic-pharmacodynamic analysis.

Quantitative bacteriology from biopsies was attempted but was prevented by the loss of serocellular crust. Bacterial presence was therefore analyzed dichotomously, although the prespecified statistical plan called for an ordinal ­analytic model.

A further limitation of this study is the modest sample size. These findings should be interpretated as evidence of efficacy in a controlled preclinical model and should be confirmed in future studies utilizing additional animals.

## CONCLUSIONS

In this porcine model of thermal burns, a thermoreversible hydrogel foam containing antibiotics demonstrated a reduction in bacterial colonization when compared to vehicle alone (HV), the standard of care (SS), and no treatment (CO), while also showing benefit in numerous secondary outcomes of wound healing. This novel treatment offers potential for advancement in the care and treatment of burn wounds. More research is warranted to evaluate this antibiotic-containing hydrogel product in clinical settings.

## Supplementary Material

usag049_Supplementary_Data

## Data Availability

The data underlying this article are available in the article and in its online supplementary material.

## INSTITUTIONAL ANIMAL CARE AND USE COMMITTEE

Animal studies were performed in compliance with the applicable federal, state, and agency laws, policies, and institutional guidelines for animal protection. This study was approved by Institutional Animal Care and Use Committee (IACUC) on January 19, 2023 (protocol number 143-2023) and approved by U.S. Army’s Animal Care and Use Review Office (ACURO) on January 23, 2023 (protocol MT21002.002.e001). The use of animals for this research complied with all ARRIVE guidelines.

## INSTITUTIONAL CLEARANCE

Submitted for institutional approval to the Public Affairs Office.
