# Supplementary material for: A Novel Foam-Forming Hydrogel Containing Antibiotics Reduces the Degree of Bacterial Colonization and Improves Wound Healing in Thermal Burns When Compared to Silver Sulfadiazine
Source: Mil Med. 2026 Aug 6;191(Suppl 1):160–7. doi: 10.1093/milmed/usag049 (PMC13445618; doi:10.1093/milmed/usag049)
Supplement: Supplementary file 1 [file usag049_supplementary_data.zip › Table S3.docx]

Exploratory Analysis of Quantitative Bacterial Growth- Pseudomonas Aeruginosa

| **Comparison** | **Odds Ratio** | **95% CI** | **Posterior Pr(OR<1)** |
| --- | --- | --- | --- |
| HA vs. CO | 1.15 | 0.39-3.41 | 39% |
| HA vs. SS | 1.38 | 0.46-4.19 | 27% |
| HV vs. CO | 1.84 | 0.63-5.53 | 10% |
| HV vs. SS | 2.20 | 0.74-6.75 | 5% |
| SS vs. CO | 0.84 | 0.28-2.53 | 64% |

**Table S3. Clinical interpretation of quantitative *Pseudomonas aeruginosa* growth by treatment.** Odds ratios with 95% CIs compare bacterial burden between groups; values <1 indicate less *P. aeruginosa* growth for the first-listed treatment. Posterior probabilities convey the likelihood of a true reduction. Clinically, HV shows greater bacterial growth vs SS, suggesting the vehicle alone performs worse than silver sulfadiazine for this bacterial strain. Other contrasts (HA vs CO, HA vs SS, HV vs CO, SS vs CO) are inconclusive with wide CIs and modest posterior probabilities, indicating no clear advantage among these pairs on this endpoint.

**Abbreviations:** CO, control; SS, silver sulfadiazine; HV, hydrogel vehicle; HA, hydrogel with antibiotic; OR, odds ratio; CI, confidence interval; Pr(OR<1), posterior probability that OR < 1.
