# Supplementary material for: A Novel Foam-Forming Hydrogel Containing Antibiotics Reduces the Degree of Bacterial Colonization and Improves Wound Healing in Thermal Burns When Compared to Silver Sulfadiazine
Source: Mil Med. 2026 Aug 6;191(Suppl 1):160–7. doi: 10.1093/milmed/usag049 (PMC13445618; doi:10.1093/milmed/usag049)
Supplement: Supplementary file 1 [file usag049_supplementary_data.zip › Figure S2.pdf]

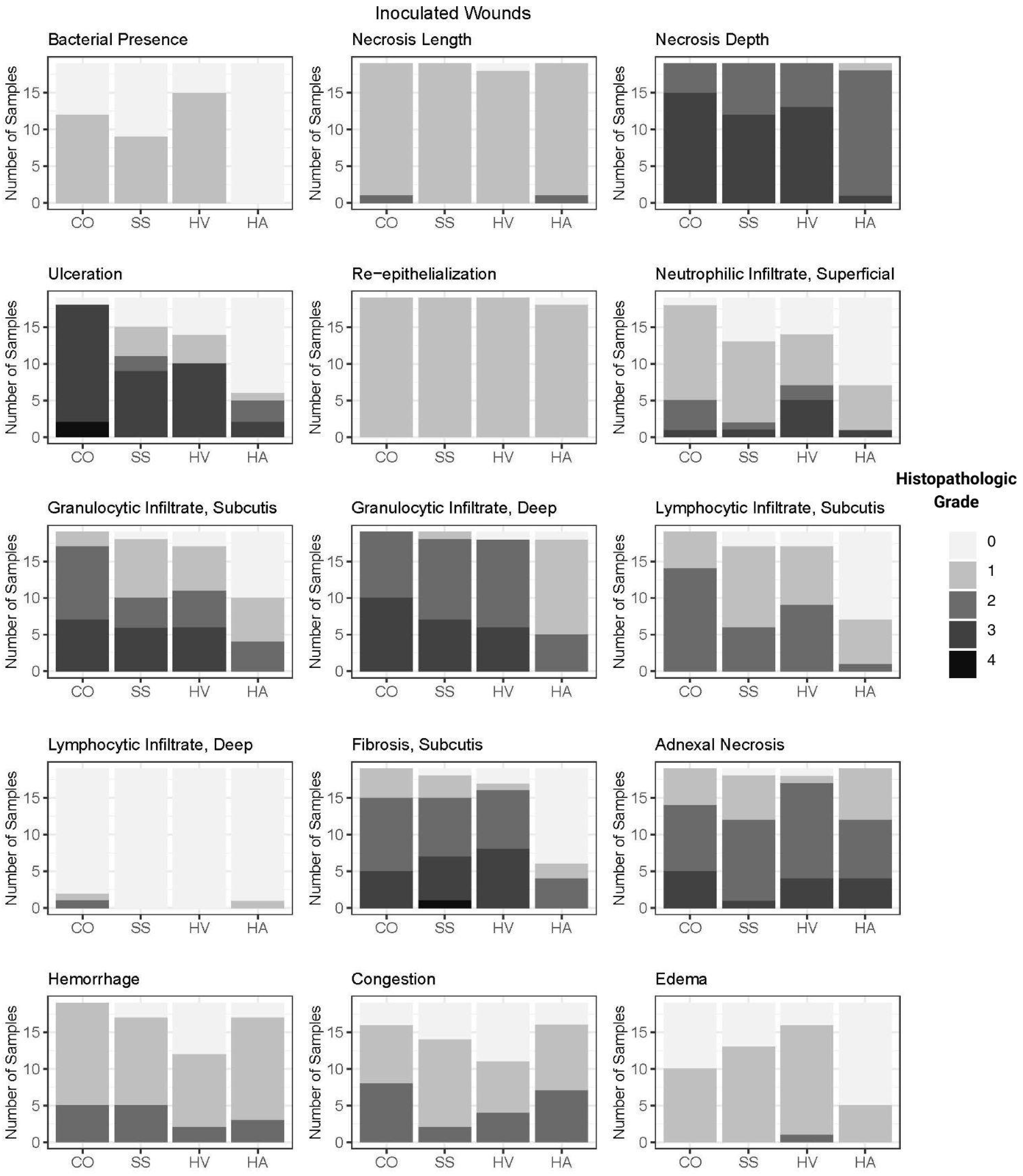

Figure S2. Histopathology distributions for inoculated wounds by treatment. Stacked bar charts display the **number of samples** at each histopathologic **grade (0–4)** for inoculated wounds across treatment groups (**CO**, **SS**, **HV**, **HA**). Outcomes include bacterial presence; necrosis length and depth; ulceration; re-epithelialization; neutrophilic infiltrate (superficial); granulocytic and lymphocytic infiltrates (subcutis and deep); fibrosis (subcutis); adnexal necrosis; hemorrhage; congestion; and edema. Darker shading indicates higher grades; legend at right provides grade mapping.

Abbreviations: CO, control; SS, silver sulfadiazine; HV, hydrogel vehicle; HA, hydrogel with antibiotic.
