# Supplementary material for: A Novel Foam-Forming Hydrogel Containing Antibiotics Reduces the Degree of Bacterial Colonization and Improves Wound Healing in Thermal Burns When Compared to Silver Sulfadiazine
Source: Mil Med. 2026 Aug 6;191(Suppl 1):160–7. doi: 10.1093/milmed/usag049 (PMC13445618; doi:10.1093/milmed/usag049)
Supplement: Supplementary file 1 [file usag049_supplementary_data.zip › Table S1.docx]

Primary Analysis: Bacterial Colonization on Histopathology

| **Comparison** | **Odds Ratio** | **98.4% CI** | **Posterior Pr(OR<1)** |
| --- | --- | --- | --- |
| HV vs. CO | 0.7 | 0.3 – 1.5 | 89% |
| HV vs. SS | 1.3 | 0.7 – 2.8 | 21% |

**Table S1. Clinical comparison of histopathologic bacterial colonization with HV versus standard care.** Odds ratios (OR) with 98.4% CIs compare HV to CO and SS for the presence of bacteria on histopathology; values <1 indicate fewer bacteria with HV. Posterior probabilities [Pr(OR<1)] summarize the likelihood of a clinically meaningful reduction. Overall, HV performs similarly to CO and SS on this endpoint, suggesting no clear antibacterial advantage of the vehicle alone in routine use.
 **Abbreviations:** CO, control; SS, silver sulfadiazine; HV, hydrogel vehicle; OR, odds ratio; CI, confidence interval; Pr(OR<1), posterior probability that OR < 1.
