# Supplementary material for: A Novel Foam-Forming Hydrogel Containing Antibiotics Reduces the Degree of Bacterial Colonization and Improves Wound Healing in Thermal Burns When Compared to Silver Sulfadiazine
Source: Mil Med. 2026 Aug 6;191(Suppl 1):160–7. doi: 10.1093/milmed/usag049 (PMC13445618; doi:10.1093/milmed/usag049)
Supplement: Supplementary file 1 [file usag049_supplementary_data.zip › Table S2.docx]

Exploratory Analysis of Quantitative Bacterial Growth- Staphylococcus Aureus

| **Comparison** | **Odds Ratio** | **95% CI** | **Posterior Pr(OR<1)** |
| --- | --- | --- | --- |
| HA vs. CO | 0.29 | 0.08-0.91 | 99% |
| HA vs. SS | 0.71 | 0.02-2.53 | 72% |
| HV vs. CO | 0.52 | 0.17-1.55 | 91% |
| HV vs. SS | 1.30 | 0.39-4.35 | 31% |
| SS vs. CO | 0.41 | 0.12-1.25 | 96% |

**Table S2. Clinical interpretation of quantitative *Staphylococcus aureus* growth by treatment.** Odds ratios (OR) with 95% CIs compare bacterial burden between groups; values <1 indicate less *S. aureus* growth for the first-listed treatment. Posterior probabilities [Pr(OR<1)] convey the likelihood of a true reduction. HA demonstrates lower bacterial growth vs CO, and SS likely reduces growth vs CO, while comparisons of HV and HA vs SS are not definitive—suggesting that the antibiotic-containing hydrogel and silver perform comparably on this endpoint and both tend to outperform control.

**Abbreviations:** CO, control; SS, silver sulfadiazine; HV, hydrogel vehicle; HA, hydrogel with antibiotic; OR, odds ratio; CI, confidence interval; Pr(OR<1), posterior probability that OR < 1.
